# Influence of acute hypoxia and radiation quality on cell survival

**DOI:** 10.1093/jrr/rrt065

**Published:** 2013-07

**Authors:** Walter Tinganelli, Ning-Yi Ma, Cläre Von Neubeck, Andreas Maier, Corinna Schicker, Wilma Kraft-Weyrather, Marco Durante

**Affiliations:** 1 Biophysics Department, GSI Helmholtzzentrum für Schwerionenforschung, 64291 Darmstadt, Germany; 2 International Open Laboratory, National Institute for Radiological Sciences, Inage-ku, Chiba, Japan; 3 Department of Radiation Oncology, Fudan University Shanghai Cancer Center, 200032 Shanghai, China; 4 German Cancer Consortium (DKTK) Dresden, OncoRay—National Center for Radiation Research in Oncology, Medical Faculty and University Hospital Carl Gustav Carus, Technische Universität Dresden, Fetscherstr. 74, 01307 Dresden, Germany; 5 German Cancer Research Center (DKFZ), Im Neuenheimer Feld 280, 69120 Heidelberg, Germany; 6 Miltenyi Biotec GmbH, Friedrich-Ebert-Straße 68, 51429 Bergisch, Gladbach, Germany; 7 Institute of Condensed Matter Physics, Darmstadt University of Technology, Hochschulestraße 3, 64289 Darmstadt, Germany

**Keywords:** hypoxia, LET, OER, radiosensitivity

## Abstract

To measure the effect of acute oxygen depletion on cell survival for different types of radiation, experiments have been performed using Chinese hamster ovary (CHO) cells and RAT-1 rat prostate cancer cells. A special chamber has been developed to perform irradiations under different levels of oxygenation. The oxygen concentrations used were normoxia (air), hypoxia (94.5% N_2_, 5% CO_2_, 0.5% O_2_) and anoxia (95% N_2_, 5% CO_2_). Cells were exposed to X-rays and to C-, N- or O-ions with linear energy transfer (LET) values ranging from 100–160 keV/µm. The oxygen enhancement ratio (OER) and relative biological effectiveness (RBE) values have been calculated from the measured clonogenic survival curves. For both cell lines, the X-ray OER depended on the survival level. For particle irradiation, OER was not dependent on the survival level but decreased with increasing LET. The RBE of CHO cells under oxic conditions reached a plateau for LET values above 100 keV/µm, while it was still increasing under anoxia. In conclusion, the results demonstrated that our chamber could be used to measure radiosensitivity under intermediate hypoxia. Measurements suggest that ions heavier than carbon could be of additional advantage in the irradiation, especially of radioresistant hypoxic tumor regions.

## INTRODUCTION

In clinical radiotherapy, hypoxia is a characteristic feature of locally advanced solid tumors [1, 2]. Those hypoxic tumor cells are often responsible for local recurrences [3] and a source of metastases [4, 5], resulting in a poor prognosis [6]. Reduced radiosensitivity in hypoxia was shown as far back as 1921 [7]. Howard-Flanders and Moore [8] demonstrated later that the sensitizing effect of oxygen can only be observed when oxygen is present at the time of irradiation, and that the effect is dependent on the oxygen concentration. At oxygen concentrations > 3% the full sensitizing effect is observed, while at lower concentrations, especially from 1% to 0.1%, a steep decrease in radiosensitivity is measured. The ratio of doses producing the same effect in hypoxic and oxic conditions respectively (OER) ranges from 1 to 3, moving from oxic to anoxic conditions. For a long time OER was used as a dose-modifying factor, i.e. independent from the survival level, but recent measurements indicate a reduced OER at low doses [9, 10]. It was shown for α-particles [11], neutrons [12] and heavier ions [13] that OER also depends on the linear energy transfer (LET), and a lower OER has been reported for high-LET values. Thus, high-LET radiation tumor therapy, such as carbon-ion therapy, offers the possibility of reducing the OER and inactivating hypoxic tumor cells more efficiently [14]. However, a strong reduction of the OER only occurs at LET values above 100 keV/µm [11, 15], and the C-ion spread-out-Bragg-peak struggles to reach these values, even in the distal region. Ions heavier than carbon, such as nitrogen or oxygen, could be interesting for hypoxic parts of the tumor [14, 16].

Recent improvements in tumor functional imaging methods allow the visualization of hypoxic tumor regions [17, 18], which will make adaptive treatment planning possible in the future. Great efforts are being made to include hypoxic tumor regions in treatment plans, mainly for X-rays [19], but now to some extent also for particle irradiation [20, 21]. However, systematic and biologically relevant OER measurements are still lacking for specific software development and validation. Most OER measurements in cells are done under oxygen-free (anoxic) conditions [22, 23], while measurements in intermediate hypoxic conditions are available only for X-rays. The oxygenation status of a tumor tissue is distributed over a continuum [24], and the fraction of cells with an oxygen level between 0.1 and 1% is normally categorized as hypoxic [25]. To include the oxygen effect into treatment planning and to test such plans, simulations of the realistic oxygen concentrations are necessary. A precondition for these measurements was the development of an irradiation system that allowed the simulation of different hypoxic states, and exposure with X-rays or ions in an extended volume. In this study, we established such a system, and then determined the hypoxic characteristics of two cell lines together with the influence of LET in small fields. We also reported the first data of a conditioned irradiation at intermediate values of LET (100 keV/μm) and oxygen concentration (0.5%).

## MATERIALS AND METHODS

### Hypoxia chamber, sample ring and gassing

**Fig. 1.**
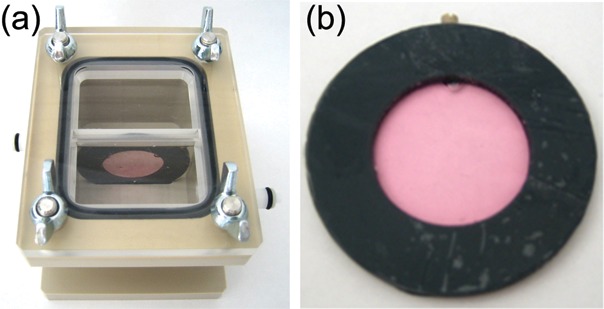
(**a**) Image of the hypoxic chamber with medium-filled sample ring. The irradiation window is facing to the front. Gas inlet and outlet are visible on both sides of the chamber. (**b**) Image of a filled sample ring. This particular ring was used for gas measurements indicated by a through boring, which is closed here with a screw.

The hypoxia chamber is made of polyetheretherketone (PEEK) and has an irradiation window with a thickness of 1 mm corresponding to a water-equivalent thickness of 1.23 mm. The top cover is transparent polymethylmethacrylate (PMMA) to allow position control of the sample. A chamfer in the bottom of the chamber and the top cover ensures the exact positioning of the sample ring (Fig. 1a). The sample ring consists of polyvinyl-chloride and has an internal diameter of 24 mm and a thickness of 3 mm (Fig. 1b). Both sides of the sample ring are covered with a gas-permeable foil of 25-μm thickness (BioFolie25, In Vitro Systems and Services, Göttingen, Germany). One layer of foil corresponds to a water-equivalent thickness of 47 µm. To achieve defined oxygen status in the chamber and samples, gassing over a gas inlet and outlet system was performed with various gas mixtures. The position of inlet and outlet guaranteed the longest possible path of the gas through the chamber to reach maximum gas exchange rates. For anoxic conditions a gas mixture of 95% nitrogen and 5%CO_2_ was used. Hypoxic conditions (intermediate oxygen concentration) were reached by gassing with 94.5% nitrogen, 0.5% oxygen and 5% CO_2_. Oxic controls were gassed with air. The gas flow was measured at the gas outlet with a flow meter calibrated for nitrogen (Vögtlin Instruments AG, Switzerland). To determine the required time and gas flow to reach the planned oxygen state in the medium, the oxygen pressure was measured using a Needle-Type housing optical O_2_ micro sensor (Pre-Sens, Regensburg, Germany). Therefore a chamber with a septum in the top cover and a sample ring with through boring at the narrow side were built to allow measurements in the cell culture medium. Determined gassing time and flow were 2 h and 200 ml/min, respectively. Measurements of the oxygen pressure were performed with empty and filled sample rings. No oxygen leaching could be measured in the range of the instrument sensibility, which is given as 0.15% at 1% oxygen concentration by the manufacturer.

### Irradiation facilities

**Fig. 2.**
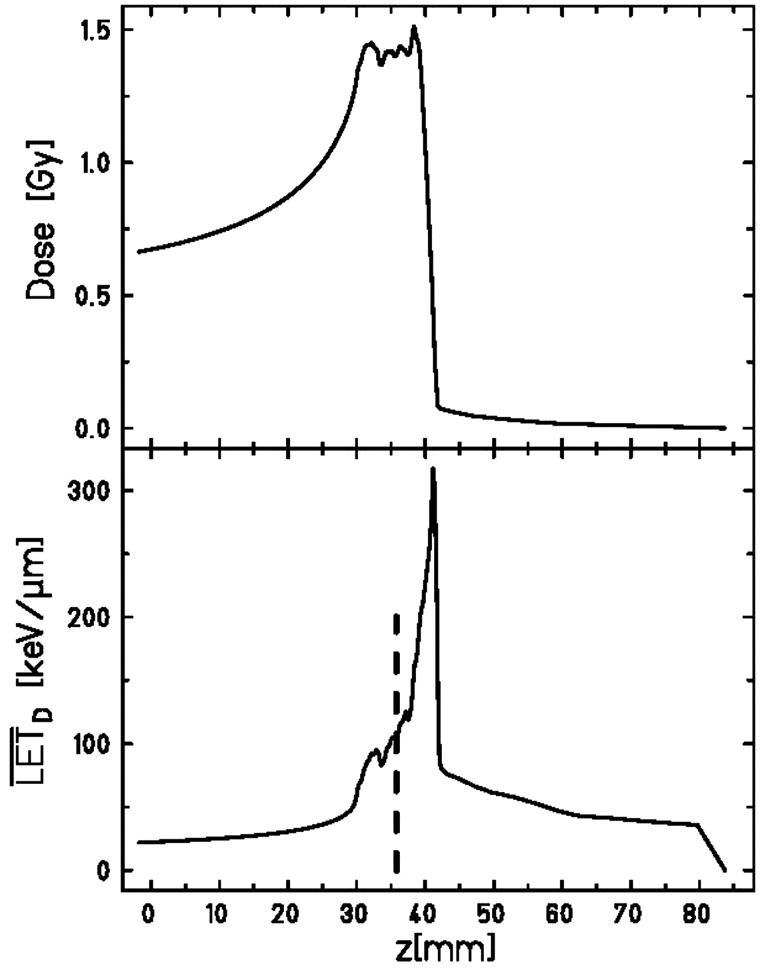
Depth dose profile of the used 1 cm SOBP (upper panel) with corresponding LET distribution (lower panel) for C-ion exposure with a dose-averaged LET of 100 keV/μm. The vertical line indicates the position of the cell layer during irradiation.

All exposures were performed through the irradiation window of the hypoxia chamber. X-ray irradiation was carried out with an Isovolt DS1 X-ray machine (Seifert, Ahrensberg, Germany) operated at 250 kVp and 16 mA with 7 mm beryllium, 1 mm aluminum and 1 mm copper filtering and a dose rate of 2 Gy/min. C-ion and N-ion irradiation was performed at the GSI synchrotron (SIS), and O-ion irradiation at the Heidelberg Ion Therapy accelerator (HIT). For all ion exposures a 1-cm spread-out Bragg peak (SOBP) was used. Figure 2 shows the profile for the carbon irradiation. The LET values are dose-average LET (LET_D_): 100 and 150 keV/μm for C-ions, 140 keV/μm for O-ions, and 160 keV/μm for N-ions.

### Cell culture and sample processing

Chinese hamster ovary K1 cells (CHO-K1) and R-3327-AT-1 (RAT-1) Dunning rat prostate cancer cells were obtained from the American tissue culture collection. CHO cells were cultured in Ham's F12 medium supplemented with 10% fetal calf serum (FCS), 50 units/ml penicillin and 50 µg/ml streptomycin (all Biochrom AG, Berlin, Germany) and incubated in a humidified atmosphere at 37ºC and 5% CO_2_. The cells had a cell cycle time of 11.0 ± 1.2 h and a plating efficiency of 0.70 ± 0.18.

The RAT-1 cells were cultivated in RPMI medium (Biochrom) supplemented with 10% FCS, 100 units/ml penicillin and 100 µg/ml streptomycin, and incubated in a humidified atmosphere at 37ºC and 5% CO_2_. The cells had a doubling time of 21 ± 2 h and a plating efficiency of 0.45 ± 0.11.

For the sample preparation, 5 x 10^4^ CHO cells or 3 x 10^4^ RAT-1 cells in 1.5 ml medium were seeded into the petri dish-like system consisting of the sample ring, which was covered on one side with the gas-permeable foil. Due to the differences in doubling time, CHO cells were incubated for 24 h while RAT-1 cells were incubated for 48 h prior to experiments. To allow gassing and exposure in a vertical sample position for ion irradiation, the ring was completely filled with medium and closed with a second foil.

Post exposure to X-rays or ions, CHO cells and RAT-1 cells were plated for a colony-forming assay. CHO cells were stained after 7 days with methylene blue while RAT-1 cells were fixed with ethanol and stained with methylene blue after 11 days. Colonies consisting of > 50 cells were considered survivors. Survival measurements for C-ions (LET 100 keV/μm) were performed three times. Data for the higher LET exposures are the result of two independent measurements. Survival curves for X-rays are the average of five or more independent experiments.

Cell cycle distribution was measured with a PASIII flow cytometer (Partec, Münster, Germany).

### Data analysis

Cell survival data were fitted with the linear quadratic model equation (eq. 1) for X-ray irradiation, and a pure exponential (eq. 2) for ion irradiation, where S is the survival level, D the dose, α the initial slope and β the curvature of survival curve:
(1)
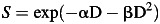

(2)
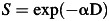



RBE values were calculated at equal survival level S according to eq. 3:
(3)
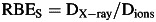



The index S indicates the survival level in percent. The RBE_α_ is the ratio of the initial slopes of the ion and the X-ray curve and represents the maximum RBE in the limit of low doses and high survival levels.

The OERs (eq. 4) were calculated accordingly, comparing the hypoxic or anoxic doses with the corresponding oxic measurements for the same ions and survival level S:
(4)
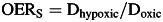



## RESULTS

### RAT-1 cells

To test the applicability of the hypoxic chamber for the irradiation of tumor cells, first measurements were performed using Dunning rat prostate cancer cells (RAT-1).

**Fig. 3.**
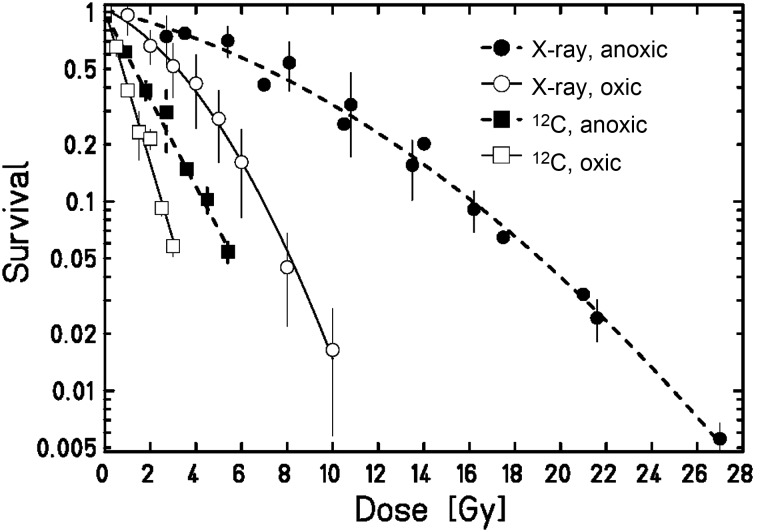
Survival of RAT-1 cells depending on oxygen concentration for X-ray and C-ion exposure with a LET_D_ of 100 keV/μm. Measurements were done under oxic (air) and anoxic (0% oxygen) conditions.

**Fig. 4.**
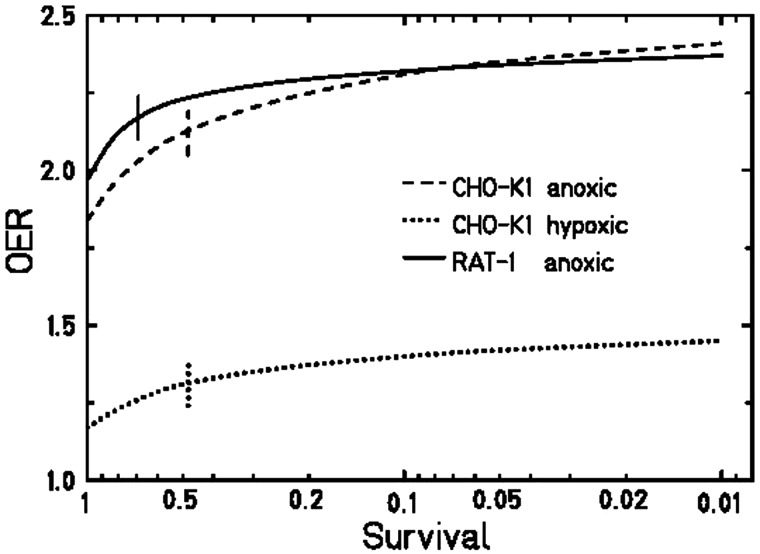
OER in dependence on cellular survival level for X-ray exposure for CHO-K1 cells under hypoxic and anoxic conditions and for RAT-1 cells under anoxic conditions. Vertical lines indicate the survival level for 50% of the total effect.

Figure 3 shows the survival of RAT-1 cells after exposure to X-rays and C-ions (LET_D_ 100 keV/μm) under oxic (air) and anoxic (0% oxygen) conditions. In Table 1 the corresponding values for α, β, RBE_10_ and OER_10_ are summarized. For X-ray irradiation the α/β value for RAT-1 cells under oxic and anoxic conditions was calculated to be 4.69 and 14.68, respectively. The OER_10_ was 2.32 ± 0.04. The OER dependence from survival level is shown in Fig. 4. The OER increased about 20% from 1.97 (OER_α_) (calculated from the initial slopes) to 2.37 (OER_1_) (calculated at 1% survival). For C-ion-exposed samples, the fit of the curves resulted in a non-significant β-value and the curves were then fitted with a simple exponential function. Consequently, it was evident that the OER did not depend on survival and was 1.77 ± 0.12 for all survival levels. RBE_10_ values were 2.8 ± 0.2 under oxic and 3.7 ± 0.1 under anoxic conditions, respectively.

**Table 1.** Summary of *α, β, RBE*_*10*_ *and OER*_*10*_ values

cell line	radiation	oxic state	α	β	*RBE*_10_	*OER*_10_
RAT-1	X-ray	oxic	0.136 ± 0.044	0.029 ± 0.004		
		anoxic	0.069 ± 0.018	0.0047 ± 0.001		2.32 ± 0.04
	C-100	oxic	0.936 ± 0.056		2.8 ± 0.2	
		anoxic	0.528 ± 0.046		3.7 ± 0.1	1.77 ± 0.13
CHO-K1	X-ray	oxic	0.164 ± 0.006	0.020 ± 0.001		
		hypoxic	0.140 ± 0.017	0.0079 ± 0.0010		1.40 ± 0.04
		anoxic	0.089 ± 0.01	0.0027 ± 0.0005		2.31 ± 0.08
	C-100	oxic	0.810 ± 0.012		2.60 ± 0.07	
		hypoxic	0.639 ± 0.042		2.88 ± 0.21	1.27 ± 0.09
		anoxic	0.409 ± 0.024		3.03 ± 0.19	1.98 ± 0.12
	C-150	oxic	0.791 ± 0.043		2.54 ± 0.14	
		anoxic	0.605 ± 0.016		4.48 ± 0.15	1.31 ± 0.08
	N-160	oxic	0.781 ± 0.024		2.51 ± 0.08	
		anoxic	0.588 ± 0.062		4.35 ± 0.46	1.33 ± 0.15
	O-140	oxic	0.707 ± 0.026		2.27 ± 0.09	
		anoxic	0.50 ± 0.12		3.72 ± 0.86	1.40 ± 0.33

### CHO-K1 cells

#### Dependence on oxygenation state

**Fig. 5.**
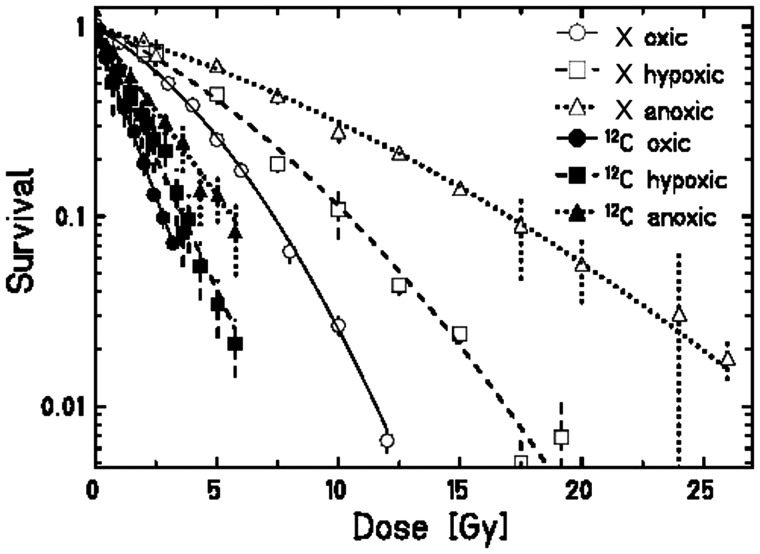
Survival of CHO-K1 cells depending on oxygen concentration for X-ray exposure, and C-ion exposure with a LET_D_ of 100 keV/μm. Measurements were done under oxic (air), hypoxic (0.5% oxygen) and anoxic (0% oxygen) conditions.

The complete set of experiments was performed using CHO-K1 cells. Figure 5 shows the survival data for CHO cells irradiated with X-rays or C-ions (100 keV/μm) under oxic (air), hypoxic (0.5% oxygen) and anoxic (0% oxygen) conditions. Table 1 summarizes the corresponding values for α, β, RBE_10_ and OER_10_. The α/β ratio after X-rays increased from 8.2 for oxic cells, through 17.7 for hypoxic, to 33.0 for anoxic cells. The OER_10_ under anoxic conditions was 2.31 ± 0.08, and under hypoxia it was reduced to 1.4 ± 0.04. Also, for CHO cells, the OER depends on survival level: under hypoxia it increased from 1.17 (OER_α_) to 1.45 (OER_1_) (24%); under anoxia from 1.84 to 2.41 (31%). The dependence of the OER on survival level is shown in Fig. 4.

Survival curves in C-ion exposures were purely exponential, leading to constant OER values. The OER_10_ under anoxic conditions was 1.98 ± 0.12, and under hypoxia 1.27 ± 0.04, resulting in RBE_10_ values of 2.60 ± 0.07 under normoxia, 2.88 ± 0.21 under hypoxia, and 3.03 ± 0.19 under anoxia.

#### LET-dependence

**Fig. 6.**
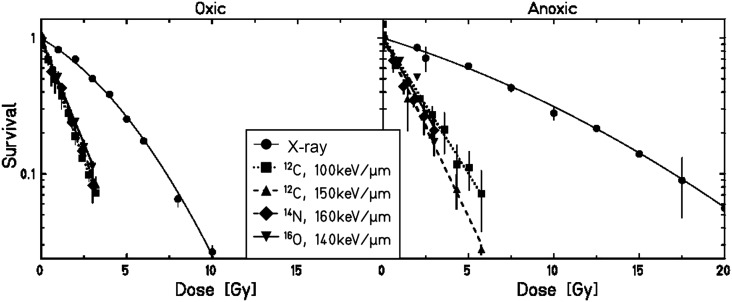
Survival of CHO-K1 cells under oxic (left panel) or anoxic (right panel) conditions depending on particle and LET in comparison with X-ray-exposed samples under the same conditions.

To measure the influence of higher LET values, experiments with C-ions (LET_D_ 150 keV/μm), N-ions (LET_D_ 160 keV/μm) and O-ions (LET_D_ 140 keV/μm) under oxic and anoxic conditions were performed. Figure 6 shows the survival as a function of particle and LET compared to X-rays under oxic and anoxic conditions, respectively. Under oxic conditions the survival curves are identical for all ions. For the anoxic conditions, a small increase in the slope of the curves was observed with increasing LET. This was reflected in the corresponding OER_10_ and RBE_10_ values (see Table 1). The RBE values under oxic conditions were already plateaued in this LET range, but there was still an increase in RBE with LET for the anoxic measurements.

## DISCUSSION

We developed an irradiation chamber that allowed the exposure of monolayer cells under different states of oxygenation to X-rays and accelerated charged particles. For this proof-of-principle experiment we used two cell lines with different origins and growth properties. For both cell lines we successfully demonstrated the dose-modulating effect of reduced oxygen conditions with both high- and low-LET radiation. Additional adhesion tests were done with human primary fibroblasts (AG1522) and a human neuroblastoma cell line (Lan-1 WT). Both cell lines grew in the sample ring system, indicating the possibility of measuring OERs for the more clinically relevant human cell lines.

### 

#### OER and oxygenation state

All exposures were done with a small, extended Bragg-peak. Survival of CHO cells to X-rays was measured under oxic, hypoxic (0.5% O_2_) and anoxic conditions. The OER_10_ for full anoxia was 2.31 ± 0.08, which is in the same range as values reported for this cell line in the literature [26, 27]. For hypoxia it was reduced to 1.40 ± 0.04. A similar systematic behaviour of the OER from the oxygenation state was found for particle exposure. Irradiation with C-ions having an LET_D_ of 100 keV/µm reduced the OER under anoxia to 1.98, and under hypoxia to 1.27. In both X-ray and C-ion irradiation the hypoxic values were ∼ 30% over the oxic values (31% for X-rays, 27% for carbon ions). For these calculations the respective anoxic values were taken as 100%. The resulting RBE_10_ values were 2.6 (oxic), 2.88 (hypoxic) and 3.03 (anoxic). Similar results were measured for V79 cells [28].

For RAT-1 cells the OER for X-rays under anoxic conditions was 2.32 ± 0.04 at 10% survival, which is in the same range as reported in [29] for four human tumor lines. For C-ions (100 keV/µm) the OER was reduced to 1.77 ± 0.12 (∼ 40%). Compared to CHO, for anoxic RAT-1 cells a higher effectiveness was measured for the carbon irradiation at this LET. RBE_10_ values for RAT-1 were 2.8 (oxic) and 3.7 (anoxic). This is an expected trend as radioresistant cells and tissues generally have smaller α/β ratio and show increased RBE values for high-LET radiation [30, 31]. Our data show that the increase in RBE for radioresistant cells was more pronounced for anoxic than for oxic cells.

#### OER and survival level

Our measurements confirm the assumption that the OER depends on survival level, especially for low doses corresponding to high survival levels. In Fig. 4 the OER for all measurements show this trend, but for RAT-1 cells the difference is smaller than for CHO cells, and restricted to very high survival levels only. From the initial OER_α_ to OER_1_ at 1% survival level there is a 20% increase, and half of this increase is reached already at a survival level of 0.68. In contrast to the RAT cells, for CHO cells the increase in OER is slower but yields greater asymptotic values: for anoxia an increase of 31% is observed, reduced to 24% under hypoxic conditions. For these cells, half of the increase in both cases is reached at a survival level of 0.48. As all samples were gassed for the same time and the range of irradiation sometimes differed, the influence of the gassing time or the time between gassing and irradiation can be excluded.

A recent review [32] of data published between 1975 and 2010 describes conflicting OER trends with survival reported for X-ray experiments. Nevertheless, the shift from a dose-modifying to dose-depending OER seems to be a real biological phenomenon and not an experimentally induced artifact [9].

An increase in OER from 2.35 at 80% survival to 2.86 at 1% for asynchronously growing CHO cells, but no dependence from survival level for synchronous cells has been reported [26]. OER was highest for S-phase cells and lowest for G1 cells. Asynchronously growing cells showed for low doses an OER similar to that in G1 which increased with decreasing survival levels to a value near that of S-phase cells. The slightly different behavior of our two measured cell lines may then be due to differences in the cell cycle distribution: RAT-1 cells had an averaged cell cycle distribution of 58% G1, 22% S and 20% G2/M, while CHO were 34% in G1, 50% in S and 16% in G2/M [33]. The higher number of S-phase cells could thus explain the greater difference in OER values for CHO cells.

#### OER and LET

As expected, we found a decrease in the OER with increasing LET. For the oxic curves, the survival in our LET region was very similar, leading to a broad maximum for RBE_oxic_, which corresponded with measurements reported for this cell line in [34]. Under anoxia, survival decreased with increasing LET, leading to a higher RBE_anoxic._ This was also reported in [28] where the maximum for RBE_oxic_ was found to be ∼150 keV/µm, whereas the reported RBE_anoxic_ increased up to 200 keV/µm. As the survival curves at these LET values could be regarded as linear under oxic as well as under anoxic conditions, OER did not change with the survival level.

**Fig. 7.**
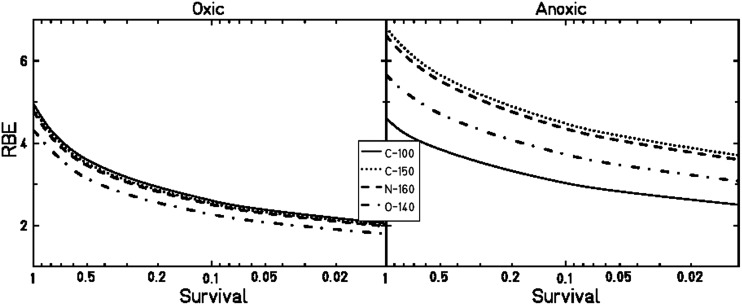
RBE in dependence on cellular survival level for CHO-K1 cells under oxic (left panel) and anoxic conditions (right panel).

Figure 7 shows the dependence of RBE for CHO cells on survival level. In consequence of the steep increase in the OER with X-rays at low doses, the RBE values under anoxic and under oxic conditions for carbon (100 keV/µm) irradiation were in a similar range for survival levels >70%. RBE values in the same range for oxic and anoxic V79 cells were reported for dose-averaged LET values below 50keV/µm [28]. For the higher LET values, RBE_anoxic_ in our measurements always exceeded RBE_oxic_. The RBE for RAT-1 cells for the carbon irradiation was higher under anoxic than under oxic conditions, even for very small doses. This may have been due to the smaller change in OER with survival level, as well as to the lower α/β ratio of these cells.

With the limited range of ion species used, a negligible effect on particle type-dependence was observed, since all data fit well with the overall LET-dependence curve measured at HIMAC [28]. There, a slight but sensible deviation in the OER versus LET profile when passing from C-ions to Ne-ions was shown, while a stronger deviation was evident for light ions (e.g. helium). A dependence on the OER on particle had already been noted in experiments in Berkely [35] comparing irradiation with C-, Ne- and Ar- ions. The ions used in the present experiments were all in the same atomic number range, so track structure effects, if any, were very difficult to assess with high statistical significance.

The measured data indicate that a clear influence of the OER on the RBE exists for C-ions in the LET range in an extended volume. This was expected, especially in the low-dose range for radioresistant tumors with a low α/β ratio. This is the dose range used in fractionated irradiation and is therefore relevant for therapy. Moreover, the cell cycle distribution within the tumor may play a major role at these doses. Heavier ions like nitrogen and oxygen could extend the range of tumor types benefiting from a reduced OER, thus producing a higher RBE for hypoxic tumor parts. Their use should, however, be carefully balanced against the risk of increased normal tissue complications, including second cancers [36].

In summary, the reported experiments demonstrated the applicability of our new hypoxic chamber for measurements with X-rays as well as with ions. It will therefore be used in future tests for adaptive treatment-planning calculations, including extended target irradiation. It has further been shown that ions heavier than carbon could be of interest for the irradiation of radioresistant hypoxic tumor parts.

## FUNDING

The work was in part supported by EU PARTNER Project No. 215840 and HGS-HIRe.
